# Exploring the mutational landscape of genes associated with inherited retinal disease using large genomic datasets: identifying loss of function intolerance and outlying propensities for missense changes

**DOI:** 10.1136/bmjophth-2022-001079

**Published:** 2022-08-25

**Authors:** Alexander Tanner, Hwei Wuen Chan, Elena Schiff, Omar A Mahroo, Jose S Pulido

**Affiliations:** 1 Ophthalmology, University College London Institute of Ophthalmology, London, UK; 2 Ophthalmology, Moorfields Eye Hospital City Road Campus, London, UK; 3 University College London Institute of Ophthalmology, London, UK; 4 Department of Ophthalmology, University of Singapore, Singapore; 5 Moorfields Eye Hospital City Road Campus, London, UK; 6 Medical Retina Service, Moorfields Eye Hospital NHS Foundation Trust, London, UK; 7 Wills Eye Hospital, Philadelphia, Pennsylvania, USA

**Keywords:** genetics, retina, dystrophy

## Abstract

**Background:**

Large databases permit quantitative description of genes in terms of intolerance to loss of function (‘haploinsufficiency’) and prevalence of missense variants. We explored these parameters in inherited retinal disease (IRD) genes.

**Methods:**

IRD genes (from the ‘RetNet’ resource) were classified by probability of loss of function intolerance (pLI) using online Genome Aggregation Database (gnomAD) and DatabasE of genomiC varIation and Phenotype in Humans using Ensembl Resources (DECIPHER) databases. Genes were identified having pLI ≥0.9 together with one or both of the following: upper bound of CI <0.35 for observed to expected (o/e) ratio of loss of function variants in the gnomAD resource; haploinsufficiency score <10 in the DECIPHER resource. IRD genes in which missense variants appeared under-represented or over-represented (Z score for o/e ratio of <−2.99 or >2.99, respectively) were also identified. The genes were evaluated in the gene ontology Protein Analysis THrough Evolutionary Relationships (PANTHER) resource.

**Results:**

Of 280 analysed genes, 39 (13.9%) were predicted loss of function intolerant. A greater proportion of X-linked than autosomal IRD genes fulfilled these criteria, as expected. Most autosomal genes were associated with dominant disease. PANTHER analysis showed >100 fold enrichment of spliceosome tri-snRNP complex assembly. Most encoded proteins were longer than the median length in the UniProt database. Fourteen genes (11 of which were in the ‘haploinsufficient’ group) showed under-representation of missense variants. Six genes (*SAMD11*, *ALMS1*, *WFS1*, *RP1L1*, *KCNV2*, *ADAMTS18*) showed over-representation of missense variants.

**Conclusion:**

A minority of IRD-associated genes appear to be ‘haploinsufficient’. Over-representation of spliceosome pathways was observed. When interpreting genetic tests, variants found in genes with over-representation of missense variants should be interpreted with caution.

**WHAT IS ALREADY KNOWN ON THIS TOPIC**

- Large genomic datasets provide metrics for individual genes relating to under-representation of predicted loss of function variants and over-representation or under-representation of missense variants.

**WHAT THIS STUDY ADDS**

- This study explores the above metrics for genes associated with inherited retinal disease: 39 were predicted ‘loss of function intolerant’ according to such metrics; 14 showed under-representation and 6 showed over-representation, of missense variants.

**HOW THIS STUDY MIGHT AFFECT RESEARCH, PRACTICE OR POLICY**

- The findings can be compared with future studies of genes associated with other disorders. Also, when interpreting genetic tests, variants found in genes with over-representation of missense variants should be interpreted with caution.

## Introduction

Inherited retinal diseases (IRDs) are a leading cause of blindness in children and the working age in many countries.^1–4^ Variants in over 250 genes are implicated. There are a number of unresolved questions relating to the spectrum of variants and mechanisms of disease.^2^ Some associated genes are ubiquitously expressed, yet pathogenic variants appear to give rise only to IRD.^5^ A number of genes show mutational hotspots, while other regions exist that rarely harbour disease-causing variants, either because the regions are highly conserved or because polymorphisms rarely cause disease. Identifying genes, or genetic regions, with particular characteristics might shed light on particular selection pressures, and also help in future interpretation of novel variants.^6 7^ The range of genes and variants involved in IRDs has been recently reviewed comprehensively by Schneider *et al*,^8^ who discussed, among other things, the prevalence of different types of variant, as well as their amenability to various gene-based therapeutic approaches.

Metrics are available from large genomic datasets which can identify those genes in which loss of function variants appear to be under-represented (conventionally termed ‘haploinsufficient’ genes).^9 10^ These metrics are an indication of those genes in which heterozygosity for loss of function variants is selected against, presumably due to a survival or molecular disadvantage.^11^ Genes can also be interrogated as to whether missense changes are significantly under-represented or over-represented. It is possible that variants that result in effects on vision, particularly if these are mild, or manifest late in life, will not have a strong effect on survival or reproductive success and so these metrics might not be affected. However, exploring these metrics for IRD genes might still yield insights into aspects of those genes in particular, potentially highlighting particularly conserved pathways, and could improve our understanding of the mutational landscape of IRD-associated genes more generally.

For this study, we curated a list of IRD genes (from the Retinal Information Network online resource, https://sph.uth.edu/retnet/), and investigated the above metrics in two large genomic databases, namely Genome Aggregation Database (gnomAD)^10^ and DatabasE of genomiC varIation and Phenotype in Humans using Ensembl Resources (DECIPHER).^12^ Both databases were used to identify genes with predicted ‘loss of function intolerance’, and the gnomAD resource was used additionally to identify those in which missense mutations were over-represented or under-represented. Genes of interest were evaluated in terms of associated pathways using the online gene ontology resource Protein Analysis THrough Evolutionary Relationships (PANTHER).^13^

The parameters investigated have been computed for each gene as a whole (based on the range of variants observed in the large datasets), rather than for any specific variants within the genes. Such parameters have been used, with some success, to identify candidate genes in whole genome data from patients with no molecular cause yet identified.^14^ In the present study, we took a converse approach: we took genes already known to be associated with retinal disease, and interrogated which of these were, in the general population, found to have an under-representation of loss of function variants, and also which had an under-representation or over-representation of missense variants.

We were interested to observe any particular patterns that emerged, estimating the proportion of IRD-associated genes classified as having an under-representation of loss of function variants and whether particular modes of inheritance were more commonly seen in this group. Similar investigations have been performed for loss of function intolerant genes in general,^15^ but our study focused in particular on IRD genes. We also explored whether such genes were more associated with syndromic disease, and whether certain pathways were over-represented. Identifying those genes with outlying propensities for missense variants could also be potentially useful: those IRD genes in which missense variants are over-represented may constitute ‘noisy genes’ such that missense variants in these genes, when found in patients, should be interpreted with caution.

## Materials and methods

### Databases and metrics

The gnomAD (https://gnomad.broadinstitute.org/) has over 141 456 individuals sequenced with 125 748 exomes and 15 708 genomes aligned against the Genome Reference Consortium Human genome build 37.^10^ Constraint variables are computed for most genes. Probability of loss of function intolerance (pLI) refers to the probability that loss of function mutations are selected against. The ratio of observed variants to the number expected (by random chance) (o/e) is also computed along with a CI. A pLI of 0.9 or greater suggests a high level of intolerance to loss of function, and this is confirmed when the CI of o/e for loss of function variants is 0.35 or lower. The o/e for missense variants can also be explored: for this study Z scores of 2.99 or greater, or −2.99 or less, were taken to indicate a significant over-representation or under-representation of missense variants (a Z value of −2.99 means that the chance of variants occurring randomly with such low frequency in the population is only 0.14% (0.0014)).

DECIPHER (https://decipher.sanger.ac.uk/)^12^ comprises genomic data from 36 000 children with rare diseases from over 270 specialist centres. Previously, a pLI separate to that from gnomAD was computed, but the pLI currently used is the gnomAD pLI. A haploinsufficiency score (HI) is also given where an index of less than 10% is taken to indicate that loss of function is significantly selected against.

### Gene classification

Genes listed in the Retinal Information Network online resource (https://sph.uth.edu/retnet/) were included in this study. Those which met both of the following criteria were identified: (1) a pLI in gnomAD of ≥0.9, and (2) an upper CI o/e limit for loss of function variants in gnomAD of <0.35 and/or an HI in DECIPHER of <10. These genes were taken as likely to be intolerant to loss of function. These genes were then evaluated in the PANTHER^13^ resource (http://pantherdb.org/) to identify common pathways in which the encoded proteins were involved, exploring which biological processes might be particularly over-represented in these gene groups (using the over-representation analysis).^16^ Also, genes with gnomAD missense (non-synonymous variants) o/e Z scores of −2.99 or less, or of 2.99 or greater, were identified and analysed similarly. The gene list curation from RetNet and investigation of metrics in gnomAD and DECIPHER were performed in September 2021; the evaluation in PANTHER was performed in February 2022.

## Results

### Intolerance of loss of function analysis

Of 309 genes and loci listed in the Retinal Information Network online resource, 29 were excluded (owing to one of the following: a mitochondrial location, no specific gene yet identified for the locus or lack of relevant data available on gnomAD and DECIPHER), leaving 280 available for inclusion (including 262 autosomal and 18 X-linked genes; online supplemental table 1). Of these, 39 genes (13.9%) met the specified criteria for loss of function intolerance. Of the IRD genes with pLI ≥0.9, there were no additional genes identified in the DECIPHER resource with an HI <10 that did not also have a gnomAD upper CI o/e limit for loss of function variants of <0.35. The 39 genes are listed in table 1. Of note, 8 of these genes are X-linked. Thus, the proportion of X-linked IRD genes fulfilling criteria for intolerance to loss of function (8/18) was 44.4% while the corresponding proportion of autosomal IRD genes (31/262) was significantly lower at 11.8% (p<0.0001 for difference in proportions). The majority of the 31 autosomal genes listed in table 1 are associated with dominantly inherited disorders. Only three genes are associated exclusively with recessively inherited disease: of note, implication of each of these genes with retinal disease has been established only by a single report, suggesting the association may not be secure. The final column of table 1 contains comments on the strength of evidence for association with monogenic retinal disease; these will be mentioned in the Discussion section.

10.1136/bmjophth-2022-001079.supp1 Supplementary data

**Table 1:** The 39 genes which fulfilled criteria of intolerance to loss of function in one or both databases

Gene	Location	Mode of inheritance of disorders	Reported phenotypes	Comments on strength of association with retinal disease
*COL11A1*	1p21.1	Dominant	Dominant Stickler syndrome type II; dominant Marshall syndrome	
*MFN2*	1p36.22	Dominant	Dominant optic atrophy with neuropathy and myopathy; dominant Charcot-Marie-Tooth disease	
*PRPF3*	1q21.2	Dominant	Dominant retinitis pigmentosa	
*EFEMP1*	2p16.1	Dominant	Dominant drusen	
*SNRNP200*	2q11.2	Dominant	Dominant retinitis pigmentosa	
*ATXN7*	3p14.1	Dominant	Dominant spinocerebellar atrophy with macular dystrophy or retinal degeneration	
*OPA1*	3q29	Dominant	Dominant optic atrophy; dominant optic atrophy with sensorineural hearing loss	
*VCAN*	5q14.3	Dominant	Dominant Wagner disease and erosive vitreoretinopathy	
*NR2F1*	5q15	Dominant	Dominant optic atrophy with intellectual disability and developmental delay (Bosch-Boonstra optic atrophy)	
*CTNNA1*	5q31.2	Dominant	Dominant macular pattern dystrophy (butterfly-shaped pigment dystrophy)	
*RIMS1*	6q13	Dominant	Dominant cone-rod dystrophy	One family reported in detail and a single patient in a second report with a different phenotype. (Other variants reported, but lacking detailed information)
*AHR*	7p21.1	Recessive	Recessive retinitis pigmentosa	One family reported
*KIAA1549*	7q34	Recessive	Recessive retinitis pigmentosa	One report of two families
*GDF6*	8q22.1	Dominant and recessive	Recessive Leber congenital amaurosis; dominant Klippel-Feil syndrome; dominant microphthalmia	Single report of Leber congenital amaurosis
*TOPORS*	9q21.1	Dominant	Dominant retinitis pigmentosa	
*PRPF4*	9q32	Dominant	Dominant retinitis pigmentosa	
*HK1*	10q22.1	Dominant and recessive	Dominant retinitis pigmentosa; recessive nonspherocytic haemolytic anaemia; recessive hereditary neuropathy	
*KIF11*	10q23.33	Dominant	Dominant microcephaly, lymphedema and chorioretinopathy	
*TEAD1*	11p15.3	Dominant	Dominant atrophia areata/Sveinsson peripapillary degeneration	
*FZD4*	11q14.2	Dominant	Dominant FEVR	
*COL2A1*	12q13.11	Dominant	Dominant Stickler syndrome, type I; dominant bone dysplasias, developmental disorders, osteoarthritic diseases, syndromic disorders	
*CCT2*	12q15	Recessive	Recessive Leber congenital amaurosis	One report
*RB1*	13q14.2	Dominant	Dominant (or somatic) retinoblastoma; pinealoma; osteogenic sarcoma	
*OTX2*	14q22.3	Dominant	Dominant syndromic microphthalmia; combined pituitary deficiency 6; early onset retinal dystrophy and pattern dystrophy	
*FBLN5*	14q32.12	Dominant	Dominant familial age-related macular degeneration; hereditary neuropathy with or without age-related macular degeneration	
*ZNF423*	16q12.1	Dominant and Recessive	Recessive nephronophthisis; dominant Joubert syndrome	One report including two families with Joubert syndrome
*PITPNM3*	17p13.2	Dominant	Dominant cone-rod dystrophy	One report of two families
*PRPF8*	17p13.3	Dominant	Dominant retinitis pigmentosa	
*C3*	19p13.3	Dominant and recessive	Dominant susceptibility to atypical haemolytic-uraemic syndrome 5; recessive CS deficiency; polymorphisms confer risk for AMD	Association with AMD, but not proven to cause monogenic retinal disease
*PRPF31*	19q13.42	Dominant	Dominant retinitis pigmentosa	
*JAG1*	20p12.2	Dominant	Dominant Alagille syndrome	
*RP2*	Xp11.23	X-linked	Retinitis pigmentosa	
*RPGR*	Xp11.4	X-linked	Retinitis pigmentosa; cone-rod dystrophy; macular dystrophy	
*DMD*	Xp21.2-p21.1	X-linked	Duchenne muscular dystrophy	Electroretinogram may be abnormal
*RS1*	Xp22.13	X-linked	X-linked retinoschisis	
*OFD1*	Xp22.2	X-linked	Joubert syndrome; orofaciodigital syndrome 1, Simpson-Golabi-Behmel syndrome 2	
*CHM*	Xq21.2	X-linked	Choroideremia	
*PRPS1*	Xq22.3	X-linked	Retinitis pigmentosa, neuropathy, optic atrophy, deafness	
*OPN1LW*	Xq28	X-linked	Deuteranopia; blue-cone monochromacy	

A number are associated also with syndromic or non-retinal disorders. The rightmost column contains comments, for some of the genes, on the strength of association with retinal disease (including highlighting those genes where there have been only single reports). These will be considered further in the Discussion section.

The 39 genes identified as showing intolerance to loss of function were then evaluated for over-representation in biological processes using the PANTHER database. The analysis showed that the following process was enriched more than 100-fold: spliceosome tri-snRNP complex assembly (p=3.44×10^−6^, false discovery rate (FDR) of 0.0049). Other processes showing significant over-representation included visual perception, eye morphogenesis, cellular protein localisation and nervous system development (online supplemental table 2) give these results in detail).

10.1136/bmjophth-2022-001079.supp2 Supplementary data

### Exploration of size of encoded proteins

It has been noted that haploinsufficient genes are significantly longer than haplosufficient genes.^17^ From 20 394 reviewed genes on the Uniprot database (https://www.uniprot.org/),^18^ we found the median protein length was 415 amino acids. Only five of the 39 genes in table 1 encode a protein with fewer than 415 amino acids, and four of these (*RS1*, *PRPS1*, *RP2*, *OPN1LW*) are X-linked. Only one gene (*OTX2*) from table 1 was both autosomal and associated with a protein with fewer than 415 amino acids.

### Analysis by frequencies of missense mutations

The 280 genes were also classified by observed/expected frequencies of non-synonymous missense variants. Fourteen genes (5.0%) were identified in which such variants appeared to be negatively selected (under-represented). These are given in table 2. Eleven of the 14 genes had already been classified as intolerant to loss of function (table 1); only *KLHL7*, *PNPLA6* and *PRPF6* are not present in table 1. As in table 1, the majority of genes in table 2 are associated with dominantly inherited disorders. PANTHER analysis revealed >100 fold enrichment of spliceosome tri-snRNP complex assembly process (p=3.00×10^−10^, FDR of 4.76×10^–6^).

**Table 2:** Genes which fulfilled criteria of negative selection for missense variants

Gene	Location	Mode of inheritance of disorders	Phenotypes
*PRPF3*	1q21.2	Dominant	Dominant retinitis pigmentosa
*SNRNP200*	2q11.2	Dominant	Dominant retinitis pigmentosa
*NR2F1*	5q15	Dominant	Dominant optic atrophy with intellectual disability and developmental delay (Bosch-Boonstra optic atrophy)
*CTNNA1*	5q31.2	Dominant	Dominant macular pattern dystrophy (butterfly-shaped pigment dystrophy)
*KLHL7*	7p15.3	Dominant	Dominant retinitis pigmentosa
*HK1*	10q22.1	Dominant and recessive	Dominant retinitis pigmentosa; recessive nonspherocytic haemolytic anaemia; recessive hereditary neuropathy
*KIF11*	10q23.33	Dominant	Dominant microcephaly, lymphedema and chorioretinopathy
*COL2A1*	12q13.11	Dominant	Dominant Stickler syndrome, type I; dominant bone dysplasias, developmental disorders, osteoarthritic diseases, syndromic disorders
*PRPF8*	17p13.3	Dominant	Dominant retinitis pigmentosa
*PNPLA6*	19p13.2	Recessive	Boucher-Neuhauser syndrome with chorioretinal dystrophy
*PRPF31*	19q13.42	Dominant	Dominant retinitis pigmentosa
*JAG1*	20p12.2	Dominant	Dominant Alagille syndrome
*PRPF6*	20q13.33	Dominant	Dominant retinitis pigmentosa
*PRPS1*	Xq22.3	X-linked	Retinitis pigmentosa, neuropathy, optic atrophy, deafness

Finally, those genes showing significantly more missense variants than expected were identified. Six genes (2.1%) met this criterion, listed in table 3. Most have long exons, and *SAMD11* has relatively poor coverage on exome sequencing analyses. All of these genes were associated with recessively inherited diseases (exclusively autosomal recessive inheritance for 5 of the 6 genes). The PANTHER pathway analysis did not find particular biological processes enriched in this small number of genes.

**Table 3:** Genes which fulfilled criteria of over-representation of missense variants

Gene	Location	Mode of inheritance of disorders	Phenotypes
*SAMD11*	1p36.33	Recessive	Recessive retinitis pigmentosa
*ALMS1*	2p13.1	Recessive	Alstrom syndrome
*WFS1*	4p16.1	Recessive	Recessive Wolfram syndrome (also autosomal dominant low frequency sensorineural hearing loss)
*RP1L1*	8p23.1	Dominant and Recessive	Dominant occult macular dystrophy; recessive retinitis pigmentosa
*KCNV2*	9p24.2	Recessive	Cone dystrophy with supernormal rod response
*ADAMTS18*	16q23.1	Recessive	Knobloch syndrome; recessive early onset retinal dystrophy

## Discussion

In this study, we explored a novel classification of retinal disease-associated genes according to metrics relating to predicted tolerance to loss of function (as defined in two large genomic databases) and to under-representation or over-representation of missense variants (as computed in the gnomAD resource). We also sought to identify any broad biological pathways that were enriched in any of these groups.

We found that approximately 14% of IRD genes (as listed in the RETnet resource) overall were predicted to be intolerant to loss of function. The proportion for X-linked genes was significantly higher than that for autosomal genes. This might be expected as the mechanism of disease for many X-linked conditions, including X-linked retinal disease is frequently loss of function.^19^ For the autosomal genes, almost all were associated with dominantly inherited conditions (and those where exclusively recessive inheritance has been described have less strong evidence for association with retinal disease). Our finding of higher proportions of autosomal dominant and X-linked (and low proportion of autosomal recessive) Mendelian diseases associated with genes with high pLI is consistent with a previous study (not focusing on retinal disease) where these genes were compared with a random sample of other genes.^15^

A number of genes encoding proteins involved in splicing complexes (*PRPF3*, *SNRNP200*, *PRPF4*, *PRPF8, PRPF31*)^20^ were found to fulfil criteria for intolerance to loss of function and are all associated with dominantly inherited disease. Of these, *PRPF31* is known to be associated with disease resulting from haploinsufficiency. Why pathogenic variants in such ubiquitously expressed genes give only a rod-cone dystrophy is still not clear: rod photoreceptors appear uniquely vulnerable to loss of function in one *PRPF31* allele. In contrast to haploinsufficiency, gain of function, often from specific missense variants, is an important mechanism in much of dominant disease. The appearance of genes in which gain of function causes retinal disease in table 1 might suggest that loss of function adversely affects survival in a way other than by affecting the retina; heterozygosity for loss of function might have severe consequences for other systems. Many, but not all, of the genes in table 1 are also associated with syndromic or non-retinal disease. We also found that the majority of IRD genes identified as intolerant to loss of function encoded proteins above the median length in terms of amino acids.

Eight of the 31 autosomal genes have associations with monogenic retinal disease that arise from reports of relatively few families, suggesting less strong evidence for causative association. The first implication of *RIMS1* in retinal disease came from a report of a single large family.^21 22^ A later report described a patient with the same *RIMS1* variant, but a different phenotype (retinitis pigmentosa),^23^ and there have further variants reported, but without detailed evidence to secure a causative relationship.^24–26^ Implication of *AHR* in retinal disease comes from a report of a single family.^27^ For *KIAA1549*, there is a single report of two families.^28^ For *GDF6*, there is a single report of a patient with Leber Congenital Amaurosis, and in this case, both parents showed electroretinography (ERG) abnormalities.^29^ Similarly, for *CCT2*, there is a single report of Leber Congenital Amaurosis.^30^ With respect to *ZNF423*, one study reported a patient with recessive nephronophthisis (no ocular data) and two families with Joubert syndrome.^31^ For *PITPNM3*, one study reported two families^32^; a subsequent study showed the carrier frequency of the variant in the general population was high for a dominant disease.^33^ Other reports of disease associated with this gene are either isolated cases or report variants that also have high carrier frequencies.^34–37^ A critical analysis of reported variants in autosomal dominant retinal dystrophies has questioned the evidence for disease association with *PITPNM3* (and also with *RIMS1*), among other genes.^38^ Finally, while the *C3* variants are associated with AMD, evidence of specific association with monogenic retinal disease is lacking. If these eight genes are excluded from the IRD list, the proportion of autosomal IRD genes meeting loss of function intolerance criteria is then 9.1%.

Interestingly, the only genes in table 1 associated with exclusively recessive inheritance are among those with evidence only from single reports. This might suggest that in cases where a novel genetic cause of recessive IRD is reported, with bi-allelic loss of function proposed as the disease mechanism, if that gene shows loss of function intolerance according to the criteria of the present study, such a report should be interpreted with caution.

We found that 5% of IRD genes showed significant under-representation of non-synonymous missense variants. The majority of these were also intolerant to loss of function, highlighting their importance in both terms. They were again mostly associated with dominantly inherited disorders, and a further gene encoding a splicing factor (*PRPF6*) emerged. Given that missense variants are relatively rare in these genes, any such novel variants found in patients with a consistent phenotype might be regarded as more likely to be pathogenic rather than incidental.

Only 2% of IRD genes met the criterion of significant over-representation of missense variants. These were all associated with autosomal recessive inheritance. This can make identifying the pathogenicity of novel missense variants in these genes more challenging. Fortunately, in *KCNV2*-associated retinopathy, electroretinography is pathognomonic for disease associated with this gene, facilitating judgements of pathogenicity of rare variants.^39^ On the other hand, variants in *RP1L1* give rise to a dominantly inherited occult maculopathy or a recessively inherited rod-cone dystrophy. The phenotypic features are not unique to this gene; the identification of this gene as one of those in which missense variants are over-represented is consistent with the reported highly polymorphic nature of *RP1L1*, and further supports the notion that novel variants, particularly those outside the two known hotspots for pathogenic variants, should be interpreted with great caution in terms of potential pathogenicity.^40^

We found that biological pathways relating to spliceosome complex assembly were enriched (>100 fold) in the group of retinal disease genes predicted to be loss of function intolerant. The spliceosome complex assembly pathway was also enriched in the group of genes with significant under-representation of missense variants. The importance of the splicing pathway is thus emphasised, together with the above-mentioned questions as to why only rod photoreceptors appear affected by heterozygous pathogenic variants.

A number of limitations of our study deserve mention. There can be pitfalls of relying on pLI indicators as described by other authors^41^; genes that do not meet the pLI criteria frequently may encode essential proteins where loss of function in one allele may cause dominant disease. The thresholds (criteria) used are somewhat arbitrary, but there was significant overlap between predictions from the gnomAD and DECIPHER databases. The reliance on only the PANTHER resource to investigate which pathways were over-represented might also represent a limitation. We therefore checked that similar results would be obtained from other resources. Entering the list of ‘haploinsufficient’ genes (from table 1) into the Reactome^42^ resource (https://reactome.org/ accessed 30 July 2022) similarly showed that mRNA splicing was most significantly over-represented (p=0.0002). We also entered these genes into the Molecular Signatures Database^43 44^ resource (V.7.5.1 available at http://www.gsea-msigdb.org/gsea/msigdb/index.jsp accessed 30 July 2022) to compute overlaps with other gene sets (selecting the Gene Ontology sets): the most significant overlap (after sensory perception gene sets, not unexpected for a group of IRD genes) was the ‘SPLICEOSOMAL_TRI_SNRNP_COMPLEX’ gene set (p=5.74×10^−10^). These findings support the validity of our results from the PANTHER analysis.

Our study is mainly exploratory, and many of the conclusions tentative. These metrics have been proposed to help identify candidate genes for unsolved diseases, whereas we have, conversely, applied these metrics to known disease-associated genes, with a view to further exploring the variant landscape of these genes.

## Data Availability

All data relevant to the study are included in the article or uploaded as supplementary information.
