# Supplementary material for: Exploring the mutational landscape of genes associated with inherited retinal disease using large genomic datasets: identifying loss of function intolerance and outlying propensities for missense changes
Source: BMJ Open Ophthalmol. 2022 Aug 25;7(1):e001079. doi: 10.1136/bmjophth-2022-001079 (PMC9422814; doi:10.1136/bmjophth-2022-001079)
Supplement: Supplementary data [file bmjophth-2022-001079supp001.pdf]

Supplementary Table 1. Parameters obtained from online gnomAD and DECIPHER databases relating to IRD-associated genes listed in the RetNet online resource.

Gene	Chromosomal location	gnomAD pLI	gnomAD plof o/e	gnomAD plof o/e CI	DECIPHER HI	gnomAD missense Z score
ABCA4	1p22.1	0	0.76	0.64-0.91	18.63	-0.66
ABCC6	16p13.11	0	0.83	0.67-1.03	72.89	-1.09
ABHD12	20p11.21	0	0.47	0.3-0.76	52.8	0.19
ACBD5	10p12.1	0.01	0.31	0.19-0.53	47.98	0.87
ACO2	22q13.2	0.21	0.24	0.14-0.42	9.79	2.92
ADAM9	8q11.23	0.03	0.26	0.17-0.42	58.29	1.9
ADAMTS18	16q23.1	0	0.74	0.6-0.93	48.74	-3.51
ADGRV1	5q14.3	0	0.44	0.38-0.52	25.57	0.07
ADIPOR1	1q32.1	0.66	0.18	0.08-0.47	11.69	2.82
AFG3L2	18p11.21	0	0.49	0.34-0.7	30.83	1.99
AGBL5	2p23.3	0	0.47	0.33-0.68	29.25	1.29
AHI1	6q23.3	0	0.75	0.59-0.95	11.12	-0.03
AHR	7p21.1	1	0.05	0.02-0.15	33.82	0.73
AIPL1	17q13.2	0	0.63	0.39-1.07	58.68	0.08
ALMS1	2p13.1	0	0.75	0.64-0.87	78.65	-2.99
ARHGEF18	19p13.3	0	0.3	0.2-0.46	73.49	2.25
ARL2BP	16q13.3	0	0.75	0.44-1.35	36.61	0.83
ARL3	10q24.32	0	0.56	0.3-1.1	12.85	1.16
ARL6	3q11.2	0.01	0.44	0.23-0.92	10.16	0.56
ARMS2	10q26.13	0	1.66	0.68-1.94	97.47	0.68
ARSG	17q24.2	0	0.76	0.52-1.15	66.13	0.89
ASRGL1	11q12.3	0	0.77	0.45-1.39	74.89	0.2
ATF6	1q23.3	0	0.47	0.32-0.7	26.13	0.56
ATXN7	3p14.1	0.96	0.16	0.08-0.33	17.71	-0.62
BBIP1	10q25.2	0	0.72	0.35-1.57	22.73	0.52
BBS1	11q13.5	0	0.69	0.5-0.98	40.7	0.16
BBS10	12q21.2	0	0.93	0.64-1.36	59.88	0.09
BBS12	4q27	0	0.62	0.41-0.96	80.48	0.34
BBS2	16q13	0	0.79	0.59-1.07	15.37	0.87
BBS4	15q24.1	0	0.86	0.63-1.18	25.15	-0.96
BBS5	2q31.1	0	0.55	0.35-0.89	9.96	0.85
BBS7	4q27	0	0.51	0.35-0.73	20.8	1.23
BBS9	7p14.3	0	0.63	0.48-0.84	17.13	0.4
BEST1	11q12.3	0	1.01	0.69-1.49	51.6	0.62
C12ORF65	12q24.31	0.21	0.29	0.12-0.91	75.97	-0.43
C1QTNF5	11q23.3	0.84	0	0-0.5	NA	1.16
C2	6p21.32	0	0.76	0.56-1.03	52.58	1.12
C21ORF2	21q22.3	0	0.5	0.53-1.4	73.17	-0.16
C2ORF71	2p23.2	0	0.82	0.61-1.11	84.17	-2.14
C3	19p13.3	0.9	0.21	0.14-0.3	66.19	2.75
C8orf37	8q22.1	0	0.79	0.46-1.42	54.96	-0.07
CA4	17q23.2	0	0.68	0.42-1.15	82.9	-0.12
CABP4	11q13.1	0	1	0.67-1.54	71.95	-0.52
CACNA1F	Xp11.23	0	0.32	0.23-0.45	39.96	2.6
CACNA2D4	12p13.33	0	0.82	0.66-1.01	65.4	0.75
CAPN5	11q13.5	0	0.53	0.36-0.78	56.23	0.53
CC2D2A	4p15.33	0	0.63	0.51-0.78	79.39	0.65

CCT2	12q15	1	0.07	0.03-0.23	4.82	1.47
CDH23	10q22.1	0	0.38	0.26-0.57	15.82	0.71
CDH3	16q22.1	0	0.59	0.42-0.85	56.91	0.08
CDHR1	10q23.1	0	0.98	0.74-1.31	54.73	-0.7
CEP164	11q23.3	0	0.67	0.54-0.84	29.41	0.35
CEP19	3q29	0.02	0.48	0.23-1.09	39.64	-0.14
CEP250	20q11.22	0	0.54	0.44-0.65	50.61	1.33
CEP290	12q21.32	0	0.84	0.71-0.98	13.34	0.47
CEP78	9q21.2	0	0.71	0.51-1.02	49.15	-0.58
CERKL	2q31.3	0	1.16	0.88-1.54	41.25	-0.27
CFB	6p21.32	0	0.33	0.22-0.52	56.27	1.48
CFH	1q31.3	0.86	0.2	0.13-0.32	73.44	1
CHM	Xq21.2	1	0.04	0.01-0.19	25.27	0.79
CIB2	15q25.1	0	0.55	0.3-1.09	53.95	0.52
CLCC1	1p13.3	0.01	0.33	0.2-0.57	69.69	1.1
CLN1/ PPT1	1p34.2	0	0.52	0.32-0.89	21.97	0.03
CLN3	16p11.2	0	0.59	0.39-0.91	55	-0.15
CLRN1	3q25.1	0	1.17	0.73-1.8	42.64	-0.59
CLUAP1	16p13.3	0	0.4	0.25-0.68	44.51	0.04
CNGA1	4p12	0	0.65	0.46-0.93	52.11	0.44
CNGA3	2q11.2	0	1.08	0.8-1.49	61.65	-0.04
CNGB1	16q21	0	0.93	0.75-1.15	68.86	-0.83
CNGB3	8q21.3	0	0.76	0.58-1.02	57.91	1.18
CNNM4	2q11.2	0	0.6	0.4-0.94	64.14	2.37
COD2	Xq27	0	0.75	0.56-1.02	27.36	-0.18
COL11A1	1p21.1	1	0.14	0.1-0.22	8.56	1.02
COL2A1	12q13.11	1	0.07	0.04-0.13	2.04	3.29
COL9A1	6q13	0	0.63	0.48-0.81	23.88	0.19
CORD1	18q21.1-q21.3	NA	NA	NA	NA	NA
CORD4	17q	NA	NA	NA	NA	NA
CORD8	1q23.1-q23.3	NA	NA	NA	NA	NA
CRB1	1q31.3	0	0.62	0.47-0.83	64.17	-1.28
CRX	19q13.32	0.51	0.19	0.08-0.6	56.39	0.33
CSPP1	8q13.1-q13.2	0	0.74	0.6-0.93	45.84	0.65
CTNNA1	5q31.2	0.97	0.17	0.1-0.31	2.28	3.66
CWC27	5q12.3	0	0.57	0.38-0.9	17.87	0.76
CYP4V2	4q35.2	0	0.7	0.49-1.03	70.45	-0.12
DHDDS	1p36.11	0.25	0.24	0.13-0.51	5.63	1.09
DHX38	16q22.2	0	0.45	0.34-0.61	31.05	2.67
DMD	Xp21.2-p21.1	1	0.1	0.07-0.15	0.26	-2.43
DRAM2	1p13.3	0	0.6	0.35-1.08	29.96	0.45
DTHD1	4p14	0	0.54	0.36-0.83	67.05	0.99
DYNC2I2	9q34.11	0	0.64	0.42-0.99	NA	-0.12
DYNC2H1	11q22.3	0	0.49	0.42-0.58	29.65	0.91
EFEMP1	2p16.1	1	0.03	0.01-0.15	11.29	1.82
ELOVL1	1p34.2	0.68	0.18	0.08-0.46	24.31	1.81
ELOVL4	6q14.1	0.83	0.16	0.07-0.41	28.93	1.19
EMC1	1p36.13	0	0.79	0.62-1.01	33.43	1.35
ERCC6	10q11.23	0	0.63	0.49-0.8	56.19	0.1
ESPN	1p36.31	0	0.63	0.43-0.93	46.08	-0.08
EXOSC2	9q34.12	0	0.66	0.42-1.1	18.74	0.39

EYS	6q12	0	0.69	0.58-0.83	25.63	0.32
FAM161A	2p15	0	0.72	0.52-1.01	75.75	-0.49
FBLN5	14q32.12	1	0.04	0.01-0.17	21.01	1.56
FLVCR1	1q32.3	0	0.38	0.23-0.67	50.23	0.85
FSCN2	17q25.3	0	1.01	0.7-1.48	55.98	-0.11
FZD4	11q14.2	0.97	0.07	0.02-0.31	22.13	0.73
GDF6	8q22.1	0.99	0	0-0.22	17.17	0.93
GNAT1	3p21.31	0	1.05	0.72-1.55	17.63	0.72
GNAT2	1p13.3	0	0.59	0.37-0.98	23.08	0.66
GNB3	12p13.31	0	0.92	0.63-1.38	28.89	1.05
GNPTG	16p13.3	0	0.79	0.53-1.22	80.99	-2.06
GPR125/ADGRA3	4p15.2	0.29	0.23	0.15-0.36	42.05	0.45
GPR179	17q12	0	0.66	0.53-0.82	79.16	0.84
GRK1	13q34	0	0.49	0.31-0.82	53.1	0.43
GRM6	5q35.3	0	0.85	0.63-1.17	55.29	-0.51
GUCA1A	6p21.1	0	0.72	0.4-1.42	40.08	0.37
GUCA1B	6p21.1	0	0.66	0.38-1.24	50.31	0.22
GUCY2D	17p13.1	0	0.52	0.37-0.75	64.67	0.78
HARS	5q31.3	0	0.49	0.32-0.76	19.43	1.26
HGSNAT	8p11.21-p11.1	0	0.49	0.34-0.73	68.49	0.71
HK1	10q22.1	0.91	0.19	0.11-0.33	49.12	3.23
HMCN1	q25.3-q31.1	0	0.41	0.35-0.48	29.46	0.28
HMX1	4p16.1	0.73	0	0-0.7	66.38	-0.1
HTRA1	10q26.13	0	0.43	0.26-0.75	19.96	1.05
IDH3B	20p13	0	0.85	0.59-1.26	20.66	0.42
IFT140	16p13.3	0	0.64	0.51-0.82	69.23	-0.81
IFT172	2p33.3	0	0.62	0.51-0.76	16.01	1.19
IFT27	22q12.3	0	0.81	0.49-1.41	55.96	0.22
IFT81	12q24.11	0	0.57	0.41-0.8	18.76	0.83
IMPDH1	7q32.1	0	0.45	0.3-0.7	17.38	1.69
IMPG1	6q14.1	0	1.05	0.82-1.35	68.61	-0.94
IMPG2	3q12.3	0	0.53	0.4-0.72	48.42	-0.21
INPP5E	9q34.3	0	0.37	0.22-0.68	77.32	0.51
INVS	9q31.1	0	0.69	0.53-0.92	32.04	1.07
IQCB1	3q13.33	0	0.64	0.47-0.9	22.44	0.31
ITM2B	13q14.2	0.63	0.17	0.07-0.54	20.15	1.04
JAG1	20p12.2	1	0.06	0.03-0.15	1.06	3.25
KCNJ13	2q37.1	0.01	0.41	0.21-0.86	17.84	1.93
KCNV2	9p24.2	0	1.88	1.34-1.97	44.67	-4.48
KIAA1549	7q34	0.99	0.18	0.12-0.29	82.97	-0.09
KIF11	10q23.33	1	0.04	0.01-0.12	9.01	3.27
KIZ	20p11.23	NA	NA	NA	NA	NA
KLHL7	7p15.3	0	0.4	0.26-0.64	12.47	3.91
KSS	Mitochondrial	NA	NA	NA	NA	NA
LAMA1	18p11.31-p11.23	0	0.51	0.43-0.62	60.72	-0.14
LCA5	6q14.1	0	0.41	0.27-0.65	45.14	-0.62
LHON	Mitochondrial	NA	NA	NA	NA	NA
LRAT	4q32.1	0.03	0.52	0.23-1.32	31.38	-0.02
LRIT3	4q25	0	0.95	0.65-1.42	77.16	0.31
LRP5	11q13.2	0.51	0.22	0.15-0.34	8.86	1.67

LZTFL1	3p21.31	0.06	0.29	0.16-0.57	50.24	1.65
MAK	6p24.2	0	0.89	0.66-1.21	58.36	0.4
MAPKAPK3	3p21.2	0	0.73	0.5-1.11	32.41	1.44
MCDR3	5p15.33-p13.1	NA	NA	NA	NA	NA
MCDR4	14q11.2	NA	NA	NA	NA	NA
MCDR5	19q13.31-q13.32	NA	NA	NA	NA	NA
MDDC	7p21-p15	NA	NA	NA	NA	NA
MERTK	2q13	0	0.54	0.39-0.75	61.44	0.59
MFN2	1p36.22	0.99	0.13	0.07-0.28	12.07	1.66
MFRP	11q23.3	0	0.88	0.64-1.22	61.74	-1.47
MFSD8	4q28.2	0	0.75	0.53-1.07	58.38	0.11
MIR204	9q21.12	NA	NA	NA	NA	NA
MKKS	20p12.2	0	0.83	0.56-1.28	38.6	-0.05
MKS1	17q22	0	0.76	0.57-1.04	34.89	0.49
MT-ATP6	Mitochondrial	NA	NA	NA	NA	NA
MT-TH	Mitochondrial	NA	NA	NA	NA	NA
MT-TL1	Mitochondrial	NA	NA	NA	NA	NA
MTTP	4q23	0	0.39	0.26-0.59	40.96	1.03
MT-TP	Mitochondrial	NA	NA	NA	40.96	NA
MT-TS2	Mitochondrial	NA	NA	NA	NA	NA
MVK	12q24.11	0.17	0.26	0.13-0.55	64.64	0.94
MYO7A	11q13.5	0	0.7	0.58-0.85	15.89	1.07
NBAS	2p24.3	0	0.65	0.55-0.78	58.53	-0.87
NDP	Xp11.3	0.65	0	0-0.88	3.8	0.97
NEK2	1q32.3	0	0.52	0.34-0.82	25.91	1.3
NEUROD1	2q31.3	0.77	0.11	0.04-0.51	0.94	0.23
NMNAT1	1p36.22	0.04	0.38	0.19-0.88	62.8	0.63
NPHP1	2q13	0	0.73	0.55-0.97	60.81	0.23
NPHP3	3q22.1	0	0.5	0.38-0.65	40.01	0.86
NPHP4	1p36.31	0	0.78	0.63-0.98	68.33	-0.24
NR2E3	15q23	NA	NA	NA	NA	NA
NR2F1	5q15	0.99	0	0-0.19	2.69	4.17
NRL	14q11.2	0.05	0.43	0.19-1.1	34.98	0.61
NYX	Xp11.4	0.13	0.36	0.15-1.13	58.32	2.11
OAT	10q26.13	0	0.61	0.39-0.96	31.64	0.88
OFD1	Xp22.2	0.96	0.17	0.1-0.32	70.24	0.32
OPA1	3q29	0.99	0.18	0.12-0.29	6.93	1.97
OPA2	Xp11.4-p11.2	NA	NA	NA	NA	NA
OPA3	19q13.32	0.57	0	0-1.13	71.9	0.05
OPA4	18q12.2-q12.3	NA	NA	NA	NA	NA
OPA5	22q12.1-q13.1	NA	NA	NA	NA	NA
OPA6	8q21-q22	NA	NA	NA	NA	NA
OPA8	16q21-q22.3	NA	NA	NA	NA	NA
OPN1LW	Xq28	0.98	0	0-0.26	67.35	0.47
OPN1MW	Xq28	0.04	0.73	0.29-1.78	71.79	0.75
OPN1SW	7q32.1	0.02	0.37	0.19-0.79	40.77	0.01
OR2W3	1q44	0	0.99	0.53-1.77	77.38	-0.66
OTX2	14q22.3	0.92	0.08	0.03-0.38	0.71	1.05
PANK2	20p13	0	0.61	0.4-0.97	28.84	0.18
PAX2	10q24.31	0.67	0.19	0.09-0.43	0.23	1.49
PCDH15	10q21.1	0	0.63	0.5-0.79	23.23	-1.68

PCYT1A	3q29	0	0.52	0.33-0.86	18.95	1.64
PDE6A	5q33.1	0	0.86	0.67-1.12	20.89	-0.43
PDE6B	4p16.3	0	0.92	0.72-1.19	31.25	-0.8
PDE6C	10q23.33	0	0.65	0.49-0.87	40.69	1.14
PDE6G	17q25.3	0.01	0.7	0.32-1.66	34.65	-0.03
PDE6H	12p12.3	0.01	0.88	0.39-1.81	39.72	0.19
PDZD7	10q24.31	0	0.76	0.52-1.13	40.76	-0.37
PEX1	7p21.2	0	0.5	0.38-0.66	50.89	1.14
PEX2	8q21.13	0	0.64	0.36-1.2	51.39	0.05
PEX7	6q23.3	0	0.97	0.67-1.42	31.6	0.5
PGK1	Xq21.1	0.77	0.15	0.06-0.47	2.45	0.34
PHYH	10q13	0	0.68	0.43-1.12	73.66	0.03
PITPNM3	17p13.2	1	0.09	0.04-0.21	47.8	2.01
PLA2G5	1p36.13-p36.12	0	0.85	0.48-1.57	78.31	0.27
PLK4	4q28.2	0	0.38	0.26-0.57	23.23	0.8
PNPLA6	19p13.2	0	0.48	0.36-0.64	32.35	4.35
POC1B	12q21.33	0	0.61	0.41-0.93	43.82	-0.09
POC5	5q13.3	0	0.59	0.4-0.92	70.73	0.39
POMGNT1	1p34.1	0	0.79	0.6-1.04	12.19	0.9
PRCD	17q25.1	0.02	0.65	0.29-1.58	64.9	0.3
PRD	Xp11.3-p11.23	NA	NA	NA	NA	NA
PRDM13	6q16.2	0.56	0.2	0.1-0.46	56.2	0.42
PROM1	4p15.32	0	0.8	0.62-1.05	42.13	-0.61
PROS1	3q11.1	0	0.35	0.23-0.57	42.01	0.58
PRPF3	1q21.2	1	0	0-0.07	11.61	3.85
PRPF31	19q13.42	0.98	0.12	0.05-0.3	27.79	3.05
PRPF4	9q32	1	0.09	0.04-0.23	5.52	1.99
PRPF6	20q13.33	0	0.29	0.19-0.44	34.68	4.85
PRPF8	17p13.3	1	0.11	0.07-0.18	4.91	8.28
PRPH2	6p21.1	0.12	0.29	0.14-0.67	36.43	0.1
PRPS1	Xq22.3	0.92	0	0-0.38	26.11	3.73
RAB28	4p15.33	0.02	0.36	0.19-0.76	12.5	0.38
RAX2	19p13.3	0.01	0.88	0.39-1.81	84.16	0.17
RB1	13q14.2	1	0.05	0.02-0.13	0.53	2.67
RBP3	10q11.22	0	0.4	0.26-0.65	56.69	-0.42
RBP4	10q23.33	0.52	0.19	0.08-0.59	32.94	0.78
RCBTB1	13q14.2	0	0.51	0.34-0.79	29.73	1.03
RCD1	6q25-q26	NA	NA	NA	34.93	NA
RD3	1q32.3	0	0.98	0.55-1.73	59.23	0.61
RDH11	14q24.1	0	0.83	0.53-1.34	47.73	0.55
RDH12	14q24.1	0	0.92	0.61-1.44	31.99	-0.14
RDH5	14q24.1	0	1.06	0.7-1.61	38.88	0.39
REEP6	19p13.3	0	0.84	0.49-1.5	83.06	-0.16
RGR	10q23.1	0	1.11	0.73-1.69	68.24	-0.28
RGS9	17q24.1	0	0.62	0.47-0.85	55.96	0.04
RGS9BP	19q13.12	0	0.87	0.42-1.76	74.65	0.67
RHO	3q22.1	0	0.6	0.35-1.08	2.43	0.24
RIMS1	6q13	0.99	0.19	0.14-0.28	9.51	2.01
RLBP1	15q26.1	0	0.64	0.39-1.08	33.98	-0.31
ROM1	11q12.3	0	0.59	0.33-1.1	15.19	-0.91
RP1	8q12.1	0	0.52	0.39-0.7	72.98	-0.89

RP17	17q23.2	0	0.68	0.42-1.15	82.9	-0.12
RP1L1	8p23.1	0	1.72	1.03-1.95	96.43	-10.07
RP2	Xp11.23	0.96	0	0-0.31	33.65	0.63
RP8	NA	NA	NA	NA	NA	NA
RP22	16p12.3-p12.1	NA	NA	NA	NA	NA
RP29	4q32-q34	NA	NA	NA	NA	NA
RP6	Xp21.3-p21.2	NA	NA	NA	NA	NA
RP63	6q23	NA	NA	NA	NA	NA
RP89/ KIF3B	20q11.21	0.08	0.26	0.15-0.45	22.85	3
RP9	7p14.3	0.02	0.38	0.2-0.8	65.7	0.69
RPE65	1p31.2	0	0.79	0.57-1.11	17.77	-0.24
RPGR	Xp11.4	1	0.04	0.01-0.21	70.26	1.25
RPGRIP1	14q11.2	0	0.69	0.54-0.88	59.78	0.25
RPGRIP1L	16q12.2	0	0.77	0.62-0.96	9.18	-0.11
RS1	Xp22.13	0.96	0	0.0-0.3	28.71	0.97
RTN4IP1	6q21	0	0.58	0.37-0.94	36.98	0.73
SAG	2q37.1	0	1.02	0.74-1.44	39.84	0.68
SAMD11	1p36.33	0	0.9	0.64-1.28	75.37	-3.44
SDCCAG8	1q43	0	0.56	0.41-0.78	19.88	-0.1
SEMA4A	1q22	0	0.41	0.27-0.64	53.05	0.22
SLC24A1	15q22.31	0	0.36	0.24-0.57	79.17	1.8
SLC25A46	5q22.1	0	0.55	0.34-0.91	15.47	0.16
SLC7A14	3q26.2	0.02	0.3	0.17-0.54	25.72	0.78
SNRNP200	2q11.2	1	0.04	0.02-0.08	19.63	5.94
SPATA7	14q31.3	0	0.8	0.57-1.15	75.54	0.04
SPP2	2q37.1	0	1.41	0.97-1.89	87.97	-0.39
TEAD1	11p15.3	1	0	0-0.12	3.27	1.66
TIMM8A	Xq22.1	0.65	0	0-0.87	16.92	1.15
TIMP3	22q12.3	0.63	0.17	0.07-0.53	3.47	1.83
TLR3	4q35.1	0	0.53	0.36-0.8	29.89	0.51
TLR4	9q33.1	0	0.66	0.45-0.99	7.75	0.65
TMEM126A	11q14.1	0	0.63	0.33-1.33	44.01	-0.57
TMEM216	11q12.2	0	0.66	0.34-1.37	24.22	0.49
TMEM237	2q33.1	0	0.86	0.6-1.25	49.63	0.09
TOPORS	9q21.1	1	0.11	0.05-0.24	18.11	1.05
TREX1	3p21.31	0.58	0.15	0.05-0.69	77.67	-0.82
TRIM32	9q33.1	0	0.45	0.26-0.85	26.93	0.83
TRNT1	3p26.2	0	0.5	0.3-0.88	70.25	-1.15
TRPM1	15q13.3	0	0.88	0.72-1.07	57.81	-0.13
TSPAN12	7q31.31	0.69	0.18	0.08-0.46	16.62	0.77
TTC8	14q32.11	0	0.48	0.32-0.74	23.33	-0.08
TTLL5	14q24.3	0	0.6	0.47-0.76	12.66	-0.04
TTPA	8q12.3	0	0.55	0.3-1.09	46.94	0.3
TUB	11p15.4	0	0.33	0.2-0.56	13.93	0.47
TUBGCP4	15q15.3	0	0.5	0.35-0.72	13.88	2.58
TUBGCP6	22q13.33	0	0.74	0.6-0.93	77.41	-0.94
TULP1	6p21.31	0	0.37	0.23-0.6	57.11	0.65
UNC119	17q11.2	0	0.71	0.4-1.33	26.92	0.93
USH1C	11p15.1	0	0.67	0.51-0.89	22.31	-0.87
USH1G	17q25.1	0	0.62	0.37-1.09	45.66	0.63
USH2A	1q41	0	0.44	0.38-0.52	4.17	0.07

VCAN	5q14.3	1	0.13	0.08-0.2	13.43	0.14
WDPCP	2p15	0	0.55	0.4-0.78	24.68	0.9
WDR19	4p14	0	0.42	0.31-0.56	40.57	1.5
WFS1	4p16.1	0	1.62	1.27-1.93	33.79	-4.71
WHRN	9q32	0	0.41	0.26-0.67	38.98	-0.32
ZNF408	11p11.2	0	0.52	0.34-0.82	80.49	0.71
ZNF423	16q12.1	1	0.07	0.03-0.19	4.5	2.49
ZNF513	2p23.3	0.82	0.16	0.07-0.41	23.47	0.53
