# Supplementary material for: Exploring the mutational landscape of genes associated with inherited retinal disease using large genomic datasets: identifying loss of function intolerance and outlying propensities for missense changes
Source: BMJ Open Ophthalmol. 2022 Aug 25;7(1):e001079. doi: 10.1136/bmjophth-2022-001079 (PMC9422814; doi:10.1136/bmjophth-2022-001079)
Supplement: Supplementary data [file bmjophth-2022-001079supp002.pdf]

**Supplementary Table 2. Results of evaluation of 39 genes in Table 1 for overrepresentation in biological processes using the PANTHER database.**

| | |
|---|---|
| Analysis Type: | PANTHER Overrepresentation Test (Released 20220202) |
| Annotation Version and Release Date: | GO Ontology database DOI: 10.5281/zenodo.5725227 Released 2021-11-16 |
| Analyzed List: | Client Text Box Input (Homo sapiens) |
| Reference List: | Homo sapiens (all genes in database) |
| Test Type: | FISHER |
| Correction: | FDR |

| GO biological process complete | Homo sapiens - REFLIST (20595) | Client Text Box Input (39) | Client Text Box Input (expected) | Client Text Box Input (over/under) | Client Text Box Input (fold Enrichment) | Client Text Box Input (raw P-value) | Client Text Box Input (FDR) |
|---|---|---|---|---|---|---|---|
| spliceosomal tri-snRNP complex assembly (GO:0000244) | 13 | 3 | 0.02 | + | > 100 | 3.44E-06 | 4.90E-03 |
| spliceosomal snRNP assembly (GO:0000387) | 37 | 3 | 0.07 | + | 42.82 | 5.88E-05 | 3.29E-02 |
| spliceosomal snRNP assembly (GO:0000387) | 218 | 11 | 0.41 | + | 26.65 | 3.15E-13 | 4.94E-09 |
| sensory perception of light stimulus (GO:0050953) | 221 | 11 | 0.42 | + | 26.28 | 3.64E-13 | 2.85E-09 |
| eye morphogenesis (GO:0048592) | 153 | 5 | 0.29 | + | 17.26 | 1.15E-05 | 8.60E-03 |
| camera-type eye morphogenesis (GO:0048593) | 125 | 4 | 0.24 | + | 16.9 | 1.01E-04 | 4.67E-02 |
| sensory organ morphogenesis (GO:0090596) | 271 | 7 | 0.51 | + | 13.64 | 7.95E-07 | 1.56E-03 |
| mRNA splicing, via spliceosome (GO:0000398) | 234 | 5 | 0.44 | + | 11.28 | 8.35E-05 | 4.22E-02 |

| | | | | | | | |
|---|---|---|---|---|---|---|---|
| RNA splicing, via transesterification reactions with bulged adenosine as nucleophile (GO:0000377) | 234 | 5 | 0.44 | + | 11.28 | 8.35E-05 | 4.09E-02 |
| RNA splicing, via transesterification reactions (GO:0000375) | 238 | 5 | 0.45 | + | 11.09 | 9.03E-05 | 4.29E-02 |
| visual system development (GO:0150063) | 375 | 7 | 0.71 | + | 9.86 | 6.51E-06 | 6.80E-03 |
| camera-type eye development (GO:0043010) | 324 | 6 | 0.61 | + | 9.78 | 3.34E-05 | 2.02E-02 |
| sensory system development (GO:0048880) | 381 | 7 | 0.72 | + | 9.7 | 7.21E-06 | 7.06E-03 |
| eye development (GO:0001654) | 371 | 6 | 0.7 | + | 8.54 | 7.01E-05 | 3.66E-02 |
| sensory organ development (GO:0007423) | 569 | 8 | 1.08 | + | 7.42 | 1.02E-05 | 7.99E-03 |
| sensory perception (GO:0007600) | 979 | 12 | 1.85 | + | 6.47 | 1.65E-07 | 4.32E-04 |
| protein-containing complex assembly (GO:0065003) | 1201 | 11 | 2.27 | + | 4.84 | 1.00E-05 | 8.25E-03 |
| nervous system process (GO:0050877) | 1434 | 12 | 2.72 | + | 4.42 | 8.85E-06 | 8.16E-03 |
| cellular protein localization (GO:0034613) | 1335 | 11 | 2.53 | + | 4.35 | 2.67E-05 | 1.82E-02 |
| cellular component assembly (GO:0022607) | 2320 | 19 | 4.39 | + | 4.32 | 7.33E-09 | 3.83E-05 |
| cellular macromolecule localization (GO:0070727) | 1345 | 11 | 2.55 | + | 4.32 | 2.87E-05 | 1.80E-02 |
| protein-containing complex subunit organization (GO:0043933) | 1372 | 11 | 2.6 | + | 4.23 | 3.44E-05 | 2.00E-02 |
| cellular component biogenesis (GO:0044085) | 2552 | 19 | 4.83 | + | 3.93 | 3.50E-08 | 1.37E-04 |
| macromolecule localization (GO:0033036) | 2289 | 15 | 4.33 | + | 3.46 | 9.16E-06 | 7.98E-03 |
| anatomical structure morphogenesis (GO:0009653) | 2180 | 14 | 4.13 | + | 3.39 | 2.60E-05 | 1.85E-02 |
| nervous system development (GO:0007399) | 2195 | 14 | 4.16 | + | 3.37 | 2.81E-05 | 1.83E-02 |
| system process (GO:0003008) | 2056 | 13 | 3.89 | + | 3.34 | 6.62E-05 | 3.58E-02 |

| | | | | | | | |
|---|---|---|---|---|---|---|---|
| system development (GO:0048731) | 4222 | 21 | 8 | + | 2.63 | 4.57E-06 | 5.52E-03 |
| multicellular organism development (GO:0007275) | 4564 | 22 | 8.64 | + | 2.55 | 3.74E-06 | 4.88E-03 |
| cellular component organization (GO:0016043) | 5314 | 25 | 10.06 | + | 2.48 | 5.71E-07 | 1.28E-03 |
| anatomical structure development (GO:0048856) | 5062 | 23 | 9.59 | + | 2.4 | 5.17E-06 | 5.79E-03 |
| cellular component organization or biogenesis (GO:0071840) | 5517 | 25 | 10.45 | + | 2.39 | 1.22E-06 | 2.12E-03 |
| developmental process (GO:0032502) | 5613 | 25 | 10.63 | + | 2.35 | 1.72E-06 | 2.70E-03 |
| multicellular organismal process (GO:0032501) | 6635 | 29 | 12.56 | + | 2.31 | 8.62E-08 | 2.70E-04 |
